# Optimized Fermentation of Endophytic *Bacillus* sp. WY17 and WY26 Consortium for Biocontrol of Ginseng Black Spot Disease and Its Antifungal Activity via Crude Protein Extract

**DOI:** 10.3390/microorganisms14091871

**Published:** 2026-08-23

**Authors:** Qiuyu Wang, Weihao Chen, Yuchi Zhao, Jiajing Liu, Jingyan Xu, Chunshi Wang, Qi Sun, Weiwei Dong, Wenxiu Ji

**Affiliations:** College of Agriculture, Yanbian University, Yanji 133002, China; w1391969303@163.com (Q.W.); 2024010621@ybu.edu.cn (W.C.); yuchi0000@163.com (Y.Z.); ljj008008@outlook.com (J.L.); 2025010688@ybu.edu.cn (J.X.); 2025050945@ybu.edu.cn (C.W.);

**Keywords:** ginseng, black spot disease, *Alternaria panax*, compound bacterial agent, crude protein

## Abstract

*Panax ginseng*, a high-value medicinal plant, faces substantial yield losses due to black spot disease, while conventional chemical controls cause pesticide residues and soil ecological damage, necessitating green biocontrol strategies. Here, two antagonistic strains, *Bacillus* sp. WY17 and WY26, were isolated from the surface-sterilized internal root tissues of 10-year-old ginseng. Through systematic optimization of carbon/nitrogen sources, inorganic salts, and fermentation parameters (temperature, pH, agitation, inoculum size, and duration), the optimal culture conditions were established. The optimal consortium consisted of WY17 and WY26 in a 2:1 ratio (WY17:WY26 = 2:1), which achieved an antifungal inhibition rate of 84.94% against the pathogen compared to the untreated control group (pathogen only). Mechanistic investigations revealed that the crude protein extract exerted its antifungal effect by compromising the integrity of the pathogen’s cell membrane, leading to increased permeability and leakage of intra-cellular contents, and produced cell wall-degrading enzymes (chitinase and β-1,3-glucanase), thereby inhibiting mycelial growth and spore germination. In vitro efficacy tests demonstrated that this crude protein extract performed comparably to the chemical fungicide 70% mancozeb, with no statistically significant difference observed between them (*p* > 0.05). These findings identify a promising compound biocontrol agent derived from indigenous *Bacillus* strains, offering an effective and environmentally friendly alternative for managing ginseng black spot disease.

## 1. Introduction

*Panax ginseng*, a perennial herb in the Araliaceae, is primarily cultivated as a high-value medicinal crop in countries such as China [1], South Korea, and Japan [2]. However, its cultivation frequently encounters the dual challenges of pathogenic fungal invasion and soil microecological imbalance. Notably, black spot disease caused by the fungal species *Alternaria panax* can lead to leaf necrosis, inhibition of plant growth, and yield losses of up to 30% [3]. The pathogen compromises host tissues by secreting cell wall-degrading enzymes (such as pectinase and cellulase) and produces toxins like dibutyl phthalate (DBP), which suppresses the plant defense mechanisms and exacerbates disease progression [4]. With the expansion of global ginseng cultivation, black spot disease has transitioned from a regional affliction to a worldwide threat, significantly hindering the sustainable development of the industry [5,6]. Although chemical pesticides, such as mancozeb and carbendazim, continue to be the primary method of pest control, their prolonged use has resulted in increased resistance among phytopathogenic fungus. Furthermore, the accumulation of soil pesticide residues has raised serious concerns regarding their detrimental effects on microbial diversity and human health. The challenges associated with traditional chemical control methods necessitate urgent attention [7,8,9]. In this context, environmentally friendly biological control techniques based on the genus *Bacillus* have emerged as a prominent area of research due to their diverse mechanisms of action.

*Bacillus* is a well-established biocontrol agent and has emerged as a focal point of research due to its diverse mechanisms of action. Studies have demonstrated that it achieves disease control through the following pathways: (1) Direct antagonism [10,11]: *Bacillus velezensis* 26-8 [12] inhibits *Alternaria panax* by up to 89.83% by secreting lipopeptide antibiotics (such as surfactin and fengycin) and cell wall hydrolases like chitinase. (2) Niche competition [13,14]: By rapidly seizing nutrients and spatial resources in the rhizosphere, *Bacillus* effectively suppresses the proliferation of phytopathogenic fungus. (3) Systemic resistance induction [5,15]: It activates the phenylpropanoid metabolic pathway and enhances the antioxidant enzyme system in ginseng plants, thereby strengthening physical defense mechanisms such as cell wall lignification. (4) Plant growth enhancement: *Bacillus* secretes plant hormones such as indoleacetic acid, which promotes root development and improves nutrient absorption efficiency [16]. Through these pathways, *Bacillus* can effectively manage disease occurrence, thereby reducing reliance on chemical pesticides.

However, the field stability of a single strain is highly susceptible to fluctuations in temperature and humidity, which limits its control efficacy due to reliance on a singular mechanism of action. In contrast, combinations of multiple bacterial strains can significantly enhance environmental adaptability and control effectiveness by engaging multiple antifungal pathways (such as antagonism, competition, and induced resistance) through synergistic interactions. The complementary characteristics exhibited by different strains within the compound bacterial agent can broaden their survival range in complex environments. Additionally, certain strains contribute to improving the microenvironment by secreting metabolites such as extracellular enzymes or signaling molecules, thereby facilitating the survival of other strains under adverse conditions [17].

Compared with non-endophytic microbes (e.g., soil or rhizosphere isolates), the screening and functional development of antagonistic strains of ginseng endophytic bacteria offer a distinct advantage as host-adaptive microbial agents, particularly in terms of improved colonization stability and reduced disruption of the native microbiome during biocontrol applications [18,19]. Despite the potential advantages of ginseng endophytic bacteria as host-adapted biocontrol agents, their application in managing ginseng diseases remains largely unexplored. Moreover, the possible synergistic mechanisms of multi-strain consortia—particularly those involving both membrane disruption and cell wall degradation—have not been elucidated. To address these gaps, this study isolated endophytic bacteria from 10-year-old ginseng roots, screened for antagonistic activity, and constructed a compound bacterial agent. We then optimized fermentation conditions to maximize its antifungal efficacy and investigated the underlying inhibitory mechanisms using in vitro ginseng segment models. By clarifying the dual-pathway synergistic mechanism (membrane destruction coupled with cell wall degradation), this work aims to provide a scientific basis for developing effective, residue-free biocontrol strategies that support the sustainable development of ginseng cultivation.

## 2. Materials and Methods

### 2.1. Experimental Materials

Pathogen and plant samples: The ginseng black spot pathogen (*Alternaria panax*, strain ACCC 39140) was purchased from the Agricultural Culture Collection of China and designated as AP-01. Healthy 10-year-old ginseng plants (*n* = 20) were collected from the ginseng planting base in Antu City, Yanbian Korean Autonomous Prefecture, Jilin Province, China (longitude: 128.5138° E, latitude: 43.2274° N, altitude: 570.8 m). Approximately 5 g of root tissue (including both main and lateral roots) was sampled from each plant, and the tissues were pooled for endophytic bacterial isolation. Potato dextrose agar (PDA) and nutrient agar (NA) media (composition as described in [20]) were used for pathogen culture and primary screening of antagonistic bacteria, respectively. Fermentation optimization was conducted using a basal medium (2% soluble starch, 0.5% beef extract, 1% CaCl_2_, pH 7.0).

### 2.2. Isolation and Screening of Antagonistic Bacteria

Healthy ginseng root tissues were surface-sterilized. Briefly, the roots were washed under running tap water, immersed in 75% ethanol for 30 s, soaked in 2% sodium hypochlorite solution for 5 min, and rinsed three times with sterile distilled water. The effectiveness of surface sterilization was verified by plating aliquots of the final rinse water onto NA medium and incubating at 28 °C for 48 h to confirm no microbial growth. Following surface sterilization, the ginseng root tissues were cut into approximately 0.5 cm pieces using a sterile scalpel. The pieces were then placed in a sterilized mortar with a small amount of sterile water and ground thoroughly until the root tissues became fibrous, yielding a suspension of ginseng endophytic bacteria. Distilled water was added to sequentially dilute the suspension into five concentration gradients: 10^−1^, 10^−2^, 10^−3^, 10^−4^, and 10^−5^. The resulting dilutions were plated onto NA medium for the isolation of endophytic bacteria [21]. A total of 38 bacterial isolates were obtained from the 20 ginseng root samples. Candidate strains were co-cultured with the pathogen (AP-01) on PDA plates (28 °C, 5 days) using the plate confrontation method for initial screening. The pathogen (AP-01) was activated and then punched into 7.0 mm diameter mycelial agar plugs. One plug was inoculated at the centre of a potato dextrose agar (PDA) plate. For each candidate strain, a single purified colony was picked and streaked symmetrically on the PDA surface at a distance of 2.5 cm from the central pathogen plug. Plates with only the pathogen plug (no antagonistic bacterium) served as negative controls. Each treatment was performed in three independent replicates. All plates were sealed and incubated upside down at 28 °C for 7 days. After incubation, the radial growth of the pathogen was measured in both control and treatment plates. The inhibition rate was calculated using the following formula: inhibition rate = [(Rc − Rt)/Rc] × 100%, where Rc is the control colony radius and Rt is the treatment colony radius. Subsequent re-screening was conducted utilizing the filter paper disc method. A 7.0 mm mycelial agar plug of the pathogen was placed at the center of a fresh PDA plate. Two sterile filter paper discs (diameter 7.0 mm) were symmetrically positioned 2.0 cm away from the center. Then, 5 µL of fermentation concentrate from the primarily selected antagonistic strain was pipetted onto each disc. Control plates received the same volume of sterile water on the discs instead of the bacterial concentrate. Each treatment consisted of three replicates. All plates were sealed and incubated upside down at 28 °C for 7 days. After incubation, the presence and size of inhibition zones were recorded, and the colony diameter of the pathogen in each plate was measured. The inhibition rate was calculated using the same formula as above. Based on their fast growth and high inhibition rates, strains WY17 and WY26 were selected as the most promising endophytic antagonists for subsequent experiments.

The 16S rRNA gene was amplified using universal primers 27F and 1492R (yielding approximately 1450 bp amplicons), and the PCR products were sequenced. The obtained sequences were compared against the NCBI GenBank database using the BLASTn algorithm, and percentage identity was calculated based on the percentage of identical nucleotides over the total aligned region. In parallel, physiological and biochemical tests, including the Voges–Proskauer (V-P) test, methyl red (M-R) test, starch hydrolysis test, indole test, nitrate reduction test, citrate utilization test, catalase test, and hydrogen sulfide production test, were performed to support the molecular identification. A phylogenetic tree was constructed using the neighbor-joining (NJ) method in MEGA 7 software. The evolutionary distances were computed using the Kimura 2-parameter model, and the reliability of the tree topology was evaluated by bootstrap analysis with 1000 replicates.

### 2.3. Construction and Optimization of the Compound Bacterial Agent

To evaluate the compatibility of strains WY17 and WY26, both strains were streak-inoculated crosswise onto NA solid medium using an inoculation loop. The plates were incubated at 28 °C for 5 days. If both strains grew well without forming any antagonistic zone at the intersection, they were considered compatible and suitable for mixed cultivation in subsequent experiments. To determine the optimal mixing ratio for maximizing the inhibitory effect against the pathogen, nine ratios (WY17:WY26 = 0:1, 1:1, 1:2, 1:3, 2:1, 2:2, 2:3, 3:1, and 3:2) were tested, and the optimal ratio was selected based on the inhibition rate. To evaluate the synergistic effect of the two strains, the expected inhibition rate (IRexp) under an additive effect was calculated using the formula IRexp = (IRWY17 × PWY17) + (IRWY26 × PWY26), where IRWY17 and IRWY26 are the inhibition rates of the respective single strains, and PWY17 and PWY26 represent their proportions in the mixture. The observed inhibition rate was then compared with the expected value to determine whether a positive synergistic interaction existed [22,23].

The optimization of the fermentation process involved screening six carbon sources (maltose, mannitol, lactose, soluble starch, glucose, sucrose), six nitrogen sources (peptone, urea, ammonium chloride, potassium nitrate, ammonium sulfate, beef extract), and six inorganic salts (potassium dihydrogen phosphate, copper sulfate, calcium chloride, sodium chloride, manganese sulfate, magnesium sulfate) through single-factor tests to identify the optimal medium composition. Based on the single-factor results, soluble starch, beef extract, and calcium chloride were selected as the optimal carbon source, nitrogen source, and inorganic salt, respectively. The three factors—soluble starch (A), beef extract (B), and calcium chloride (C)—were each tested at three concentrations: 1.0%, 2.0%, and 3.0% for A; 0.5%, 1.0%, and 1.5% for B; and 0.5%, 1.0%, and 1.5% for C. The strain was cultured under the nine combinations at 28 °C and 200 r/min for 48 h. Building upon these findings, additional single-factor tests were performed to evaluate different temperatures, rotational speed, inoculation size, pH, and time in order to establish optimal culture conditions. Biomass was selected as the primary optimization criterion because it is a rapid and reliable indicator of bacterial growth in *Bacillus* fermentation optimization studies [24,25], and preliminary experiments confirmed a positive correlation between OD_600_ and antifungal activity under our conditions. The fermentation broth was centrifuged at 10,000 rpm and 4 °C, and the supernatant was used to assay the activities of protease, chitinase, and β-1,3-glucanase. These enzymes were selected because they are key extracellular hydrolases associated with the antagonistic activity of *Bacillus* spp. against fungal pathogens.

### 2.4. Stability Determination and Antifungal Effect Test of the Supernatant of the Fermentation of the Compound Strain

Nutrient broth (NB) liquid medium was prepared according to the manufacturer’s instructions, containing 3 g/L beef extract, 5 g/L peptone, and adjusted to pH 7.2 ± 0.2. The bacterial strain was inoculated into NB liquid medium and incubated at 28 °C with shaking at 200 rpm for 48 h. Subsequently, the culture was centrifuged at 4 °C and 10,000 rpm for 30 min. The supernatant was then sterilized using a 0.22 µm filter membrane, after which it was collected for stability tests [26,27]. For thermal stability determination, 20 mL of the supernatant was heated in a water bath at 80 °C for 30 min. To assess enzyme stability, Proteinase K was added to the supernatant at a concentration of 2 mg/mL and incubated at 37 °C for two hours. The bacteriostatic activity was evaluated on the sterile fermentation broth subjected to these stability tests, with untreated sterile fermentation broth serving as the control group. Each treatment was repeated three times to ensure reproducibility of the results.

### 2.5. Separation of Active Substances and Detection of Antifungal Activity in the Supernatant of Fermentation of the Compound Strain

Crude protein, crude lipopeptide, and organic solvent extracts (petroleum ether, n-butanol, and ethyl acetate) were obtained from the fermentation supernatant following the protocol described in [28]. The inhibitory effect of the active substances on black spot disease was assessed using the poisoned food technique [29]. The isolated and purified active substance was incorporated into PDA medium at a ratio of 1:99. After solidification of the plates, mycelial agar plugs (7.0 mm in diameter) of the ginseng black spot pathogen were inoculated at the center of each plate; pure PDA medium without any antifungal substance served as the control. Each group underwent three repetitions, with cultures maintained at 28 °C for 10 days. Colony diameters were measured using a cross-crossing method to calculate the inhibition rate (formula: inhibition rate = [(Dc − Dt)/Dc] × 100%, Dc: control colony diameter, Dt: treatment colony diameter).

### 2.6. Inhibition of Ginseng Black Spot Pathogen by Different Concentrations of Antifungal Active Substances

From the active substances that exhibited significant antifungal effects, the optimal inhibitory concentration was determined using the poisoned food technique. The antifungal active solution was prepared by diluting with sterile water to obtain various concentrations (1, 5, 10, 15, 20, and 30 mL/L). Following the same procedures as described in Section 2.5, the inhibition rate was calculated. Based on the inhibition rates against mycelial growth at different concentrations, a virulence regression equation was established to determine the EC_50_ value of the active substance.

After culturing the ginseng black spot pathogen in PDA medium at 28 °C for ten days until spore maturation occurred, spores were collected by rinsing with sterile water while removing mycelium through gauze filtration. The spores were then diluted to a concentration of approximately 2.0 × 10^6^ spores/mL, as determined using a hemocytometer. Extracts of the active substance were prepared in methanol at varying concentrations (1, 5, 10, 15, 20 and 30 mL/L). Double concave slides were utilized; each well received an addition of 100 μL spore suspension mixed with an equal volume of active substance solution at different concentrations. An equal volume of methanol was used as a solvent control to exclude any potential effect of the solvent on spore germination. Each group consisted of three replicates and was incubated in a constant temperature chamber set to maintain conditions at 28 °C and relative humidity at 80%. After 24 h, when the spore germination rates in the control group exceeded 90%, the number of germinated spores was examined microscopically. Subsequently, the spore germination rates across different concentrations were calculated, along with the corresponding virulence regression equations and EC_50_/EC_70_ values.

For the filter paper plate confrontation method, symmetrical filter paper discs were moistened with 5 μL of the active substance from the compound bacterial agent. In contrast, the control group received an equal volume of sterile water applied to the filter paper plates. After a culture period of 7 days, mycelium adjacent to the filter paper plates was carefully extracted and rinsed with a 0.1 mol/L phosphate-buffered solution. The morphological changes in the mycelium were subsequently observed under a microscope.

### 2.7. Effects of Antifungal Active Substances on Cell Membrane Permeability

The effects of the crude protein extract on cell membrane permeability of the pathogen were evaluated by measuring electrical conductivity and the leakage of intracellular macromolecules (proteins and nucleic acids). Two separate experiments were conducted using the EC_50_ and EC_70_ concentrations of the crude protein extract, with sterile distilled water as the control.

For electrical conductivity measurement, six mycelial agar plugs (5 mm in diameter) of the pathogen were placed in each beaker containing 20 mL of distilled water, followed by the addition of 20 mL of the crude protein extract at EC_50_ or EC_70_ concentration. Electrical conductivity was monitored at 0, 1, 2, 3, 4, and 5 h post-treatment using a conductivity meter. Each treatment was performed in triplicate.

For measurement of intracellular macromolecule leakage, the mycelial plugs were first cultured in PDA liquid medium at 25 °C for three days. After incubation, the crude protein extract at EC_50_ or EC_70_ concentration was added. At 0, 1, 2, 3, 4, and 5 h post-treatment, 10 mL aliquots of the culture supernatant were collected and centrifuged at 10,000× *g* for 5 min. The absorbance of the supernatant was measured at 260 nm (for nucleic acids) and 280 nm (for proteins) using a spectrophotometer. Each treatment was performed in triplicate.

### 2.8. Effects of Antifungal Active Substances on the Physiological Metabolism of Phytopathogenic Fungus

The regulatory effects of antifungal active substances on the physiological metabolism of the ginseng black spot pathogen were analyzed using multiple indicators [16]. Enzyme activities (protease, chitinase, and β-1,3-glucanase) were measured in the mycelia of *A. panax* after treatment with the crude protein extract. The anthrone colorimetric method was employed to measure absorbance at 620 nm, with the measured content calculated based on the standard curve (y = 1.2464x + 1.5766, R^2^ = 0.9949). Absorbance was assessed at 595 nm using the Coomassie brilliant blue method, and protein content was determined according to the standard curve (y = 0.0152x + 0.1003, R^2^ = 0.9916). Additionally, absorbance measurements at 680 nm were conducted via the Folin-phenol method to determine protease activity based on a standard curve (y = 0.0103x + 0.305, R^2^ = 0.9960). The DMAB colorimetric method was utilized for measuring absorbance at 585 nm to assess chitinase activity in accordance with its standard curve (y = 0.6097x + 0.0041, R^2^ = 0.9982). Finally, β-1,3-glucanase activity was evaluated using the DNS colorimetric method by measuring absorbance at 540 nm against a standard curve (y = 0.8183x + 0.0182, R^2^ = 0.9909).

### 2.9. Ex Vivo Efficacy Assay of Antifungal Active Substances Against Ginseng Black Spot Pathogen

To evaluate the ex vivo efficacy of antifungal active substances against the ginseng black spot pathogen, healthy and undamaged fresh ginseng roots free from pathogen infection were selected for this study. Following surface disinfection treatment, cylindrical tissue blocks measuring 0.5 cm in diameter and 0.2 cm in thickness were excised [30]. Three pieces of ginseng root were placed in each Petri dish, with an appropriate amount of sterile water added to maintain moisture levels. A black spot fungus cake with a diameter of 5 mm was inoculated at the center of the tissue surface. Three days post-inoculation with the black spot pathogen, the crude protein extract and the chemical fungicide 70% mancozeb were sprayed, respectively. The 70% mancozeb was used as a commercially available wettable powder formulation (active ingredient: mancozeb 70%; inert ingredients 30%), which was dissolved in sterile distilled water and applied at the recommended field concentration (according to the manufacturer’s instructions). Lesion spread and decay were observed at 28 °C.

### 2.10. Data Analysis

For percentage data, arcsine square-root transformation was applied prior to analysis to improve normality. The results of the orthogonal array design were analyzed by range analysis to determine the optimal combination of the three factors. The EC_50_ and EC_70_ values were estimated by fitting the inhibition rate data to a nonlinear logistic regression model (sigmoidal dose–response curve) using IBM SPSS version 25.0. Data from other experiments were subjected to one-way ANOVA using IBM SPSS version 25.0, after confirming normality with the Shapiro–Wilk test and homogeneity of variances with Levene’s test, followed by Duncan’s multiple range test for post hoc comparisons. The significance level of *p* < 0.05 was considered statistically significant. Data are presented as mean ± standard deviation (*n* = 3).

## 3. Results

### 3.1. Isolation and Identification of Antagonistic Bacteria

**Figure 1:**
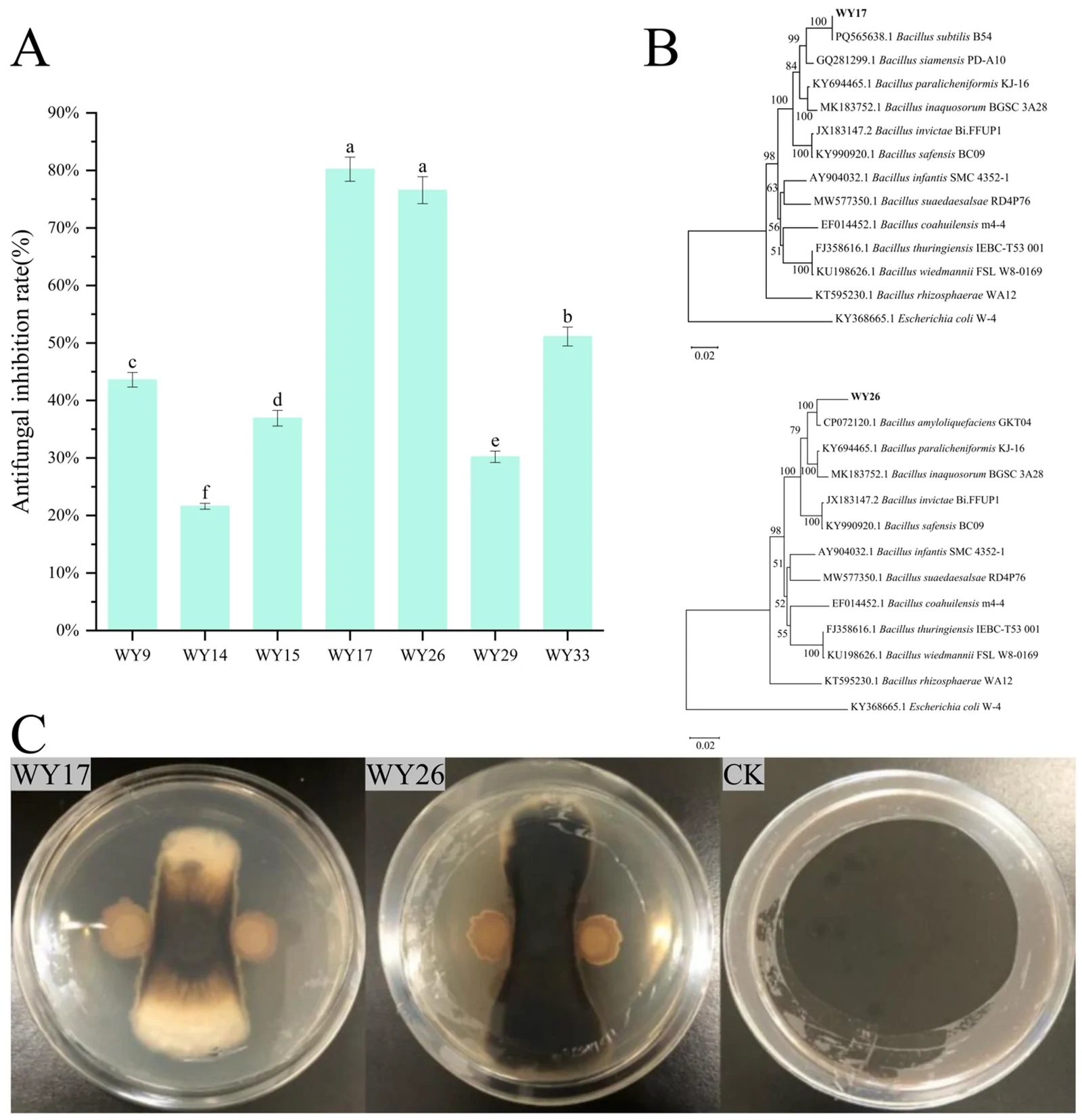
Screening and identification of antagonistic strains against ginseng black spot disease from ginseng roots. (**A**) Inhibitory effect of antagonistic strains on the black spot pathogen, (**B**) phylogenetic tree of antagonistic strains WY17 and WY26, (**C**) inhibitory effects of antagonistic strains WY17 and WY26 with control (CK). Significant differences (Tukey’s test, *p* < 0.05) are denoted by distinct lowercase letters above bars.

A total of 38 bacterial isolates were initially obtained from the root tissues of 10-year-old ginseng plants. Using the plate confrontation method, seven strains exhibiting antagonistic effects against the ginseng black spot pathogen were selected from the root tissues of 10-year-old ginseng plants. Among these, strains WY17 and WY26 demonstrated significant inhibitory effects, with antifungal inhibition rates of 80.21% and 76.56%, respectively (*p* < 0.05) (Figure 1A,C).

Physiological and biochemical assays revealed that both strains tested negative in the Voges–Proskauer (V-P) and Methyl Red (M-R) tests, could hydrolyze starch, produced red substances in the indole test, and tested positive in the nitrate reduction test. Additionally, strain WY17 tested negative in the citrate utilization test and positive in the catalase test; strain WY26 tested positive in both the catalase test and hydrogen sulfide production test (Table 1).

Based on 16S rRNA gene sequence analysis, BLASTn (version, 2.17.0) searches against the NCBI GenBank database revealed that strain WY17 (GenBank accession number PZ639072) shared 97.30% sequence identity with *Bacillus subtilis*, while strain WY26 (GenBank accession number PZ639069) shared 98.77% sequence identity with *Bacillus amyloliquefaciens*. A phylogenetic tree was constructed using the neighbor-joining method with reference sequences from type strains of closely related *Bacillus* species obtained from NCBI GenBank (Figure 1B). Due to the limited resolution of 16S rRNA gene sequencing for species-level differentiation, strains WY17 and WY26 were identified as members of the genus *Bacillus* and designated as *Bacillus* sp. WY17 and WY26.

### 3.2. Process Optimization of Compound Bacterial Strain

**Figure 2:**
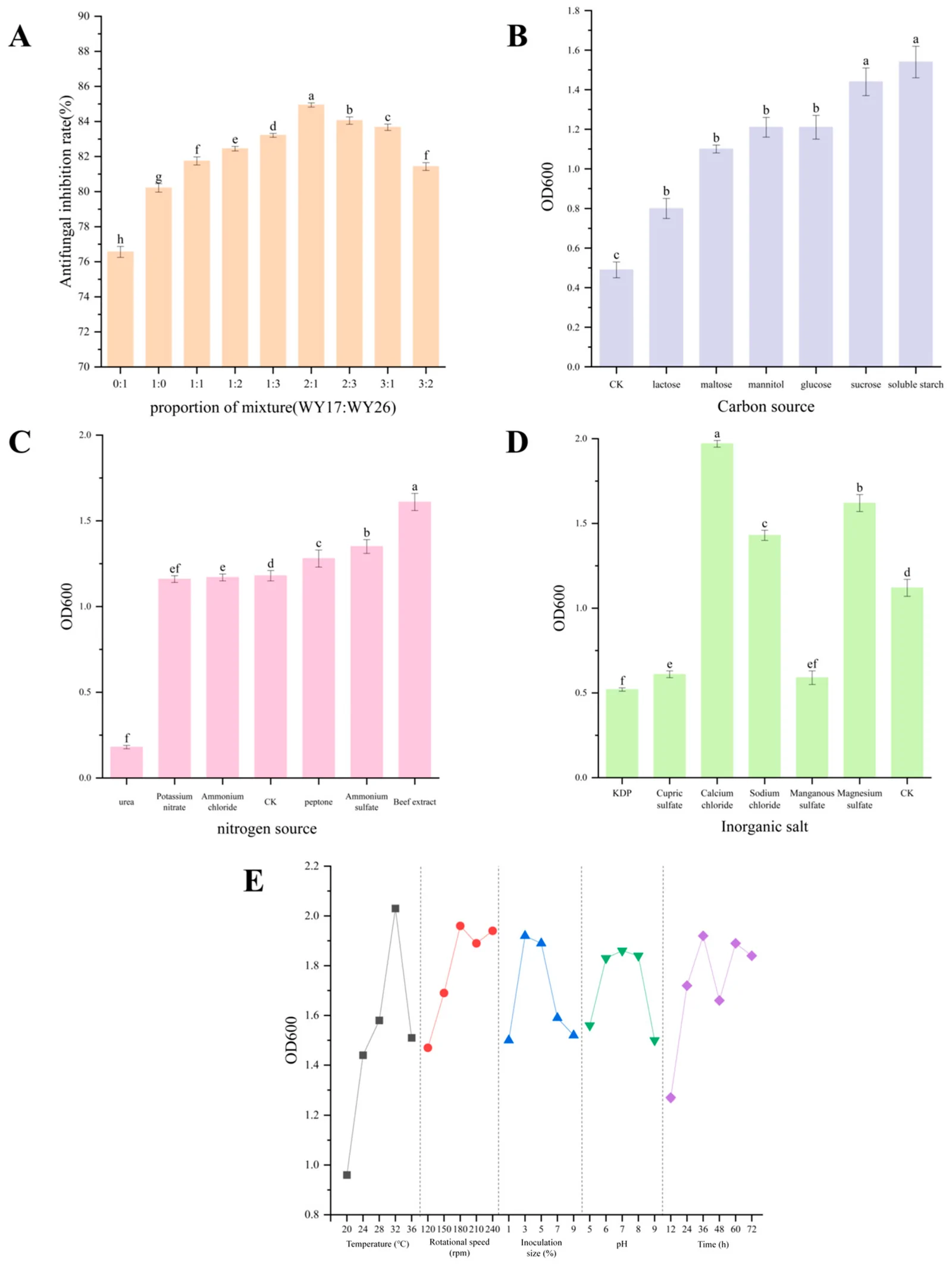
Antifungal effects of composite strains and optimization of fermentation conditions. (**A**) Inhibitory effects of different mixing ratios of composite strains, (**B**–**D**) effects of different carbon sources, nitrogen sources and inorganic salts on complex bacterial fermentation, (**E**) optimization of fermentation conditions for the composite strain. Significant differences (Tukey’s test, *p* < 0.05) are denoted by distinct lowercase letters above bars.

Cross-streaking tests confirmed that WY17 and WY26 were metabolically compatible and could be co-cultured without mutual antagonism. Nine different mixing ratios (WY17:WY26 = 0:1, 1:1, 1:2, 1:3, 2:1, 2:2, 2:3, 3:1, and 3:2) were established for the antagonistic test. The results demonstrated that when WY17 and WY26 were combined in a 2:1 ratio, the antifungal inhibition rate reached as high as 84.94% (Figure 2A), which was between 4.73% and 8.38% higher than that of individual strains (*p* < 0.05). To formally evaluate the synergistic effect, the expected inhibition rate under an additive effect was calculated as (IRWY17 × 0.667) + (IRWY26 × 0.333), yielding an expected value of 78.99%. The observed inhibition rate (84.94%) exceeded the expected rate by 5.95 percentage points, indicating a positive synergistic interaction. The inhibitory effect of the compound bacterial agent against the ginseng black spot pathogen was significantly more pronounced than that of single strains, thereby exhibiting a synergistic effect.

The optimal component types were identified from a selection of six candidate carbon sources (maltose, mannitol, lactose, soluble starch, glucose, sucrose), six nitrogen sources (peptone, urea, ammonium chloride, potassium nitrate, ammonium sulfate, beef extract), and six inorganic salts (potassium dihydrogen phosphate, copper sulfate, calcium chloride, sodium chloride, manganese sulfate, magnesium sulfate) through a single-factor test. For carbon sources, soluble starch supported the highest cell density (OD_600_ = 1.54), significantly outperforming the other five carbon sources tested. Among the nitrogen sources, beef extract gave the maximum OD_600_ value of 1.61, which was notably higher than that of the inorganic nitrogen sources. For inorganic salts, calcium chloride resulted in the greatest biomass accumulation (OD_600_ = 1.97), whereas the other salts showed relatively lower growth-promoting effects. Based on these single-factor experimental results, the optimal components were determined as soluble starch (carbon source), beef extract (nitrogen source), and calcium chloride (inorganic salt) for subsequent fermentation optimization (Figure 2B–D).

Based on these findings, using different components as factors and different addition amounts as levels, an orthogonal experiment was designed. Each factor had three levels: soluble starch addition amount: level 1 (1.0%), level 2 (2.0%), level 3 (3.0%); beef extract addition amount: level 1 (0.5%), level 2 (1.0%), level 3 (1.5%); calcium chloride addition amount: level 1 (0.5%), level 2 (1.0%), level 3 (1.5%). The concentration ratio of the aforementioned components was optimized through an orthogonal experiment L9 (3^3^). The results indicated that soluble starch (2%), beef extract (0.5%), and calcium chloride (1%) constituted the optimal medium components, significantly enhancing the biomass of the bacteria (OD600 = 1.974) (Table 2). Further single-factor experiments were conducted to optimize the culture conditions (temperature, rotational speed, inoculation size, pH, and time), and the optimal fermentation parameters were determined as 32 °C, 180 rpm, 3% inoculation, pH 7.0, and a fermentation duration of 36 h (Figure 2E).

### 3.3. Assessment of Fermentation Supernatant Stability, Separation of Active Substances, and Evaluation of Antifungal Inhibitory Effects

**Figure 3:**
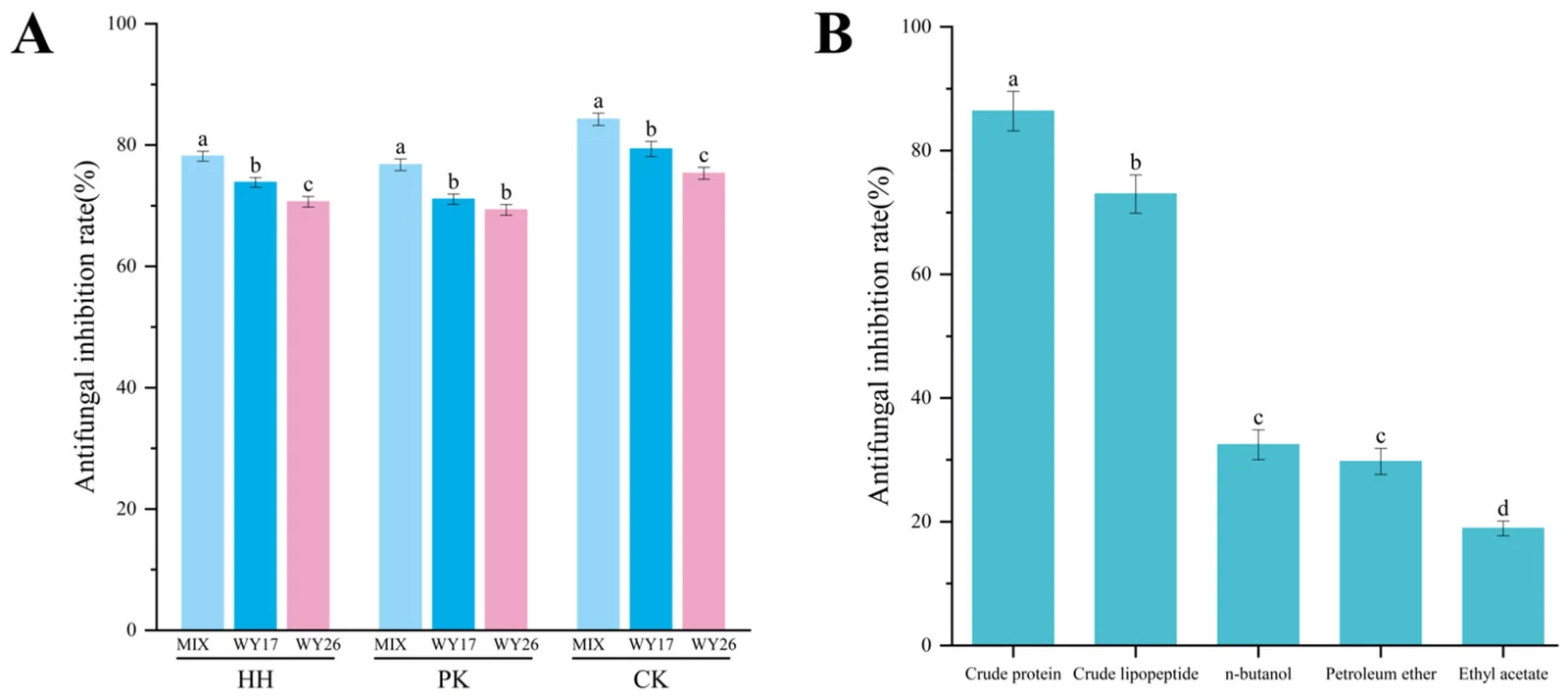
Assessment of Fermentation Supernatant Stability, Separation of Active Substances, and Evaluation of Antifungal Inhibitory Effects. (**A**) Antifungal activity and stability assessment of fermentation supernatant. CK: untreated control containing the same fermentation supernatant (without heat or Proteinase K treatment); HH: heat-treated supernatant (80 °C, 30 min); PK: Proteinase K-treated supernatant (2 mg/mL, 37 °C, 2 h), (**B**) antagonistic effects of the antibacterial active substances on black spot disease. Significant differences (Tukey’s test, *p* < 0.05) are denoted by distinct lowercase letters above bars.

In this study, we evaluated the antifungal stability of the fermentation supernatant derived from a compound bacterial agent. The results indicated that the antifungal rate of the fermentation supernatant following high-temperature treatment (80 °C, 30 min) was 78.16% ± 0.825%, which was significantly higher than that of the fermentation supernatants from individual strains WY17 (73.84% ± 0.792%) and WY26 (70.64% ± 0.882%) (*p* < 0.05). Furthermore, after treatment with Proteinase K (2 mg/mL), the inhibition rate of the fermentation supernatant remained at 76.76% ± 0.955% (Figure 3A). Under conditions of high temperature, high pressure, and Proteinase K treatment, there was a slight reduction in the antifungal inhibition rate of the fermentation supernatant. The crude protein extract retained substantial antifungal activity under these stress conditions, indicating that it has relatively stable properties against heat and protease treatment.

Crude protein, crude lipopeptide and extract were obtained through a staged separation (ammonium sulfate precipitation, acidification extraction, and organic solvent extraction). Among these components, the antifungal activity of the crude protein reached an impressive 86.37%, significantly surpassing that of the other components (*p* < 0.05) (Figure 3B).

### 3.4. The Inhibitory Effect of Different Concentrations of Crude Protein on the Ginseng Black Spot Pathogen

**Figure 4:**
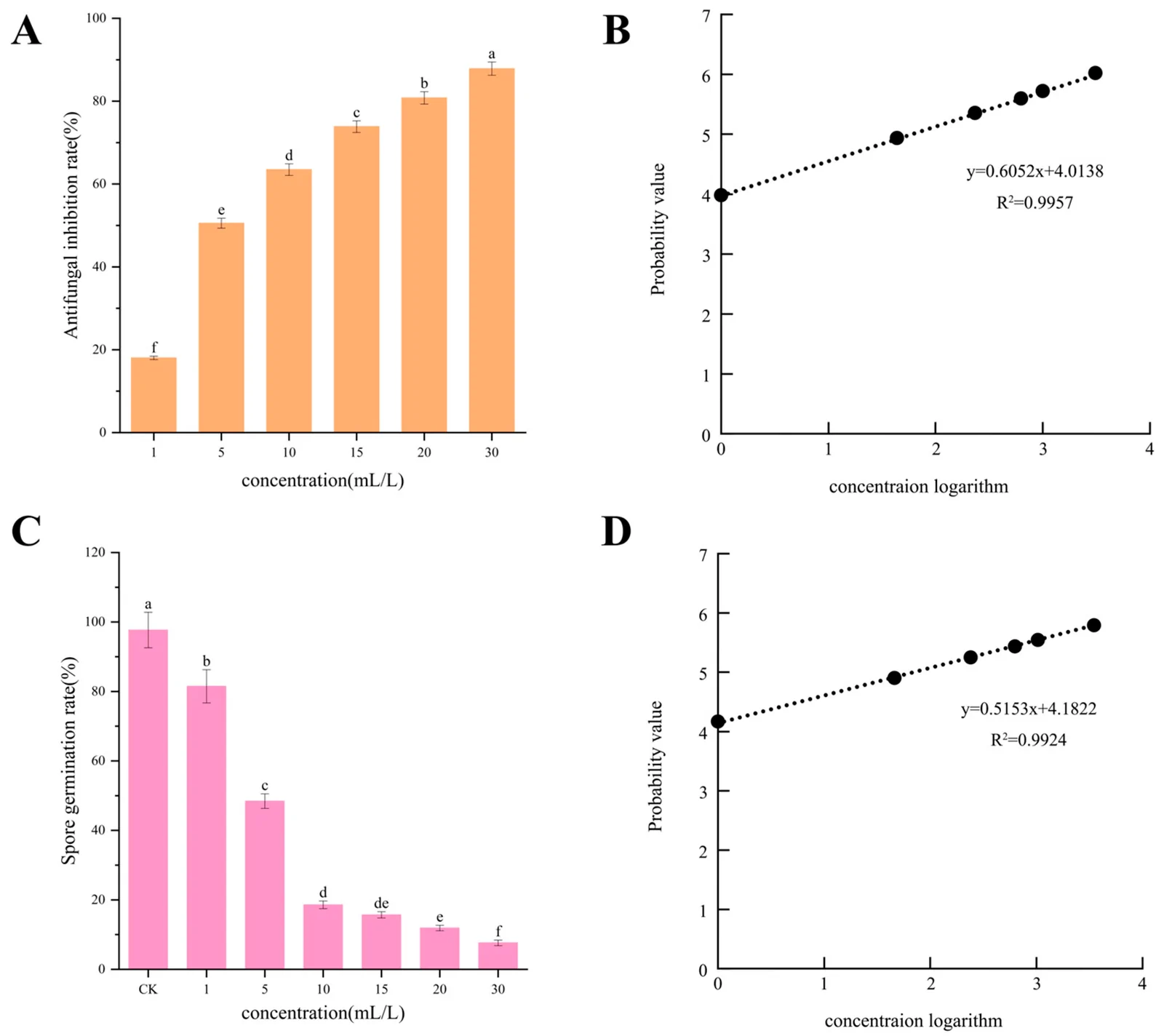
Inhibition of ginseng black spot pathogen and spore germination by compound crude protein at different concentrations. (**A**) Inhibition of ginseng black spot disease by compound crude protein at different concentrations, (**B**) virulence regression equation of compound crude protein on mycelial growth of ginseng black spot pathogen, (**C**) effect of compound crude protein at different concentrations on spore germination rate, (**D**) virulence regression equation of compound crude protein in inhibiting spore germination of black spot ginseng fungus. Data are presented as mean ± SD (*n* = 3). Significant differences (Tukey’s test, *p* < 0.05) are denoted by distinct lowercase letters above bars.

The crude protein derived from the compound bacteria exhibited a significant inhibitory effect on fungal growth, with the antifungal inhibition rate increasing in a concentration-dependent manner (*p* < 0.05) (Figure 4). At a treatment volume of 30 mL, the mycelial inhibition rate reached 87.85% (Figure 4A), and the EC_50_ value was determined to be 5.1 mL/L (Figure 4B). Furthermore, the crude protein obtained from the compound bacterial agent significantly suppressed spore germination associated with black spot disease, demonstrating an enhanced inhibitory effect as concentration increased. Under a treatment condition of 30 mL/L, the spore germination rate decreased to 7.63% (*p* < 0.05) (Figure 4C), while the EC_50_ value was calculated to be 4.9 mL/L (Figure 4D).

### 3.5. Effects of Crude Protein from Composite Strains on Hyphal Morphology and Membrane Permeability of the Pathogen

**Figure 5:**
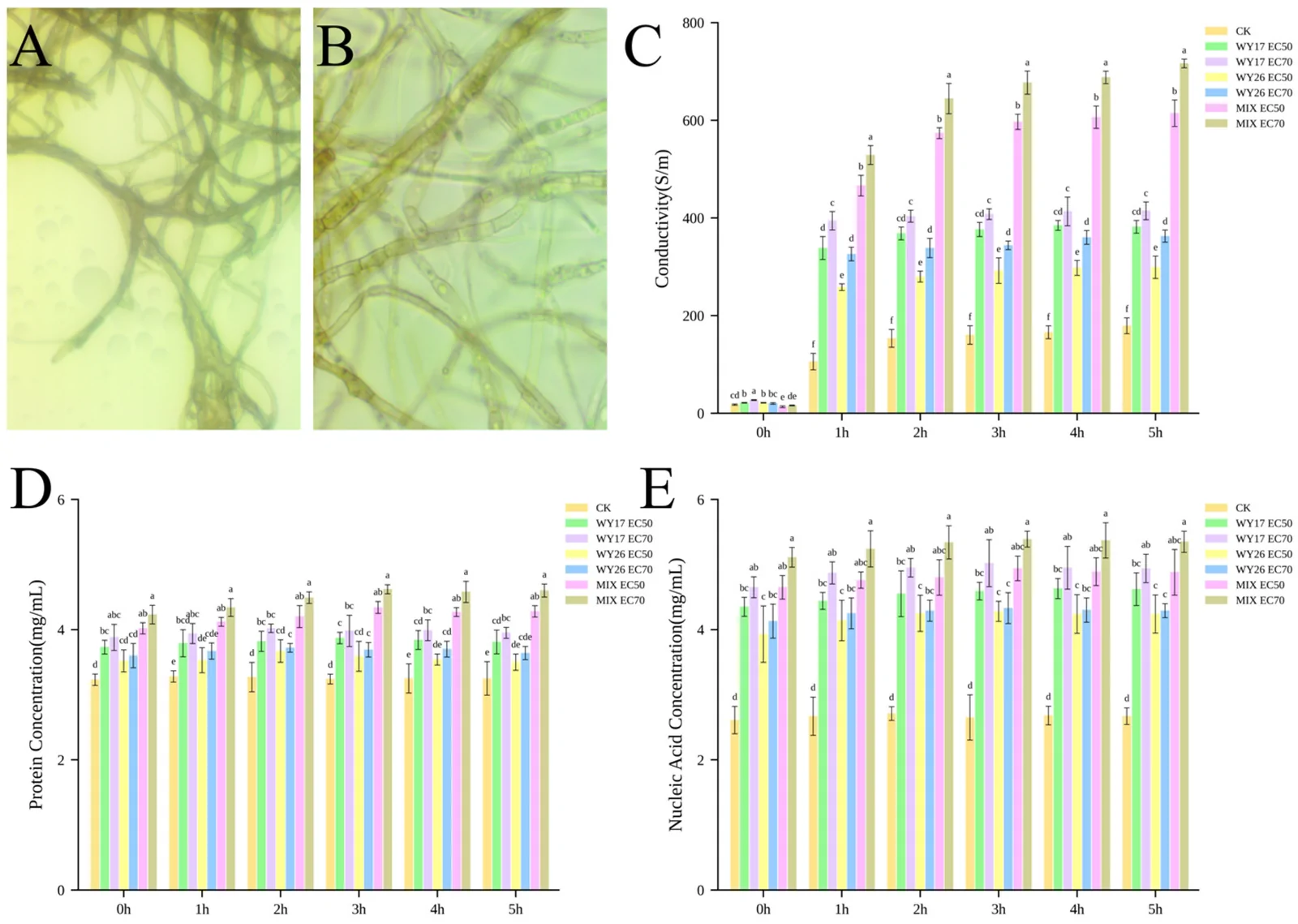
Effects of crude protein from composite strains on hyphal morphology and membrane permeability of the pathogen. (**A**,**B**) Hyphal morphology of the pathogen, (**C**) conductivity, (**D**) protein concentration, (**E**) nucleic acid concentration. Significant differences (Tukey’s test, *p* < 0.05) are denoted by distinct lowercase letters above bars.

This study systematically investigated the dual inhibitory effects of complex bacterial crude protein on the integrity of cell membranes and the metabolic functions of the black spot pathogen. Figure 5A depicts the mycelium of the black spot fungus without any treatment. The mycelium exhibits no surrounding secretion, with distinct and short septa, uniform growth, and a pronounced black coloration. In contrast, Figure 5B presents the mycelial conditions following the inhibition of ginseng’s black spot fungus by crude protein derived from the compound bacterial agent. In this figure, the mycelium of ginseng’s black spot fungus appears malformed and swollen, exhibiting a vacuolated structure. Some hyphae have ruptured; there is a reduction in branching; color has become pale; both the number of septa and their spacing have increased; and secretion is observed around the mycelium. The crude protein from the compound fungus inflicts damage on the morphological structure of ginseng’s black spot fungal mycelium, leading to abnormal growth patterns (Figure 5A,B).

Dynamic monitoring of electrical conductivity revealed that the electrical conductivity of the compound bacterial agent EC_70_ consistently increased over time. After 5 h of treatment, electrical conductivity was three times higher than that observed in the control group, indicating increased cell membrane permeability (Figure 5C).

Over time, the concentrations of protein and nucleic acid in the control group remained largely unchanged. However, following treatment with the crude protein derived from compound bacteria, a consistent trend was observed (Figure 5D,E). The concentrations of both protein and nucleic acid were significantly higher than those recorded in the control group, indicating damage to the cell membranes of the pathogen’s mycelium. Notably, the EC_70_ treatment resulted in the highest levels of protein and nucleic acid concentration.

### 3.6. The Effect of Complex Bacterial Crude Protein on the Physiological Metabolism of Black Spot Disease

**Figure 6:**
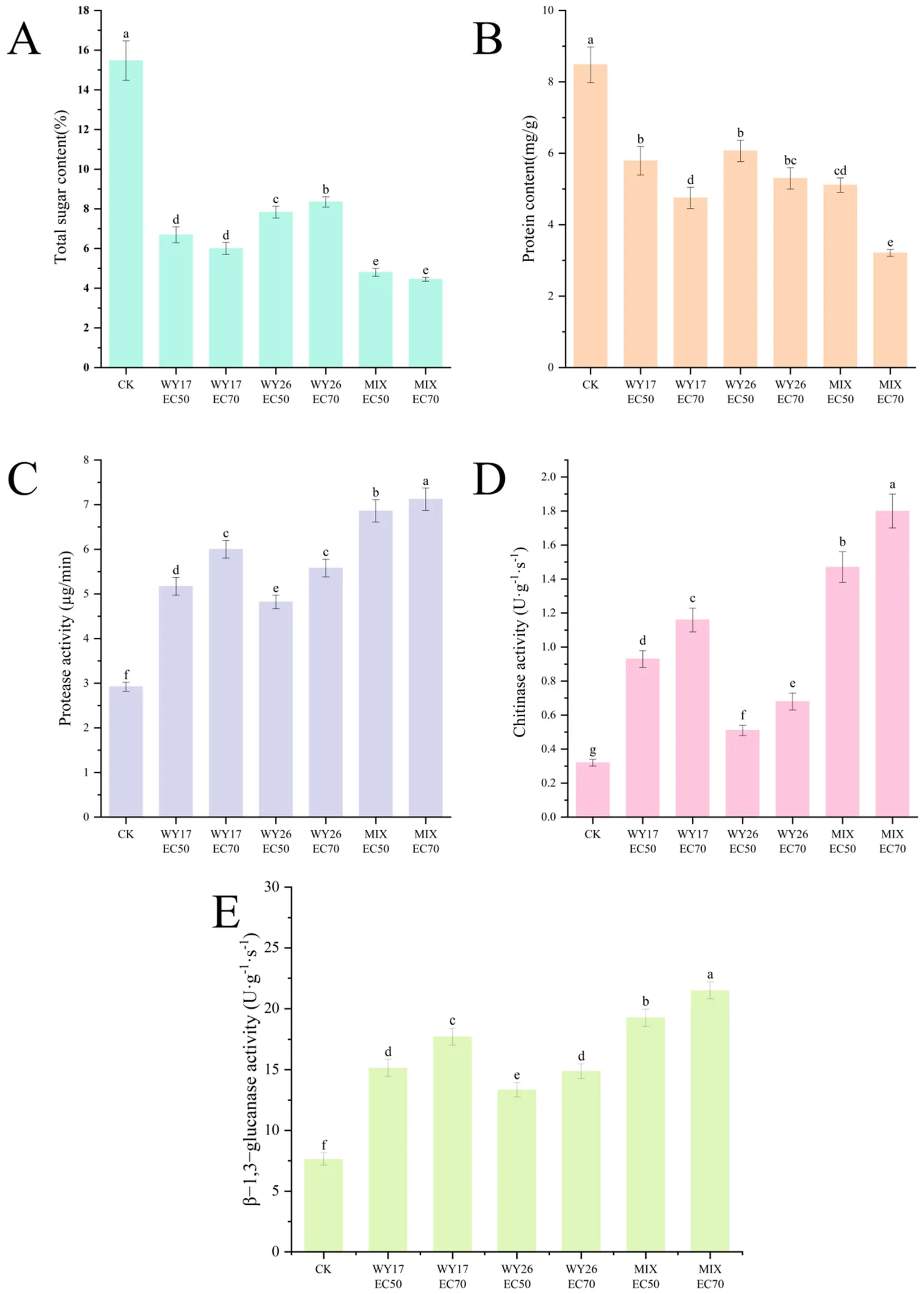
The effect of complex bacterial crude protein on the physiological metabolism of phytopathogenic fungus. (**A**) total sugar content, (**B**) protein content, (**C**) protease activity, (**D**) chitinase activity, (**E**) β-1, 3-glucanase activity. CK represents the negative control treated with sterile water. Significant differences (Tukey’s test, *p* < 0.05) are denoted by distinct lowercase letters above bars.

Further analysis of metabolic activity in the pathogen mycelia revealed that under EC_70_ treatment with the crude protein extract from the composite bacterial agent, total sugar content decreased by 71.24% and protein content by 62.12% compared to the control group. Treatment with the crude protein extract demonstrated a more pronounced effect on carbohydrate metabolism and protein synthesis as the concentration increased (Figure 6A,B).

The activities of protease, chitinase, and β-1,3-glucanase in the treatment group were significantly higher than those observed in the control group. Furthermore, the crude protein extract from the composite bacterial agent exhibited greater efficacy compared to single-strain treatments. Under EC_70_ treatment of the crude protein extract, protease activity was found to be 2.44 times that of the control group, chitinase activity was 5.57 times greater than that of the control group, and β-1,3-glucanase activity was 2.81 times higher than that of the control group. Additionally, an increase in the concentration of the crude protein extract correlated with a more rapid hydrolysis rate for protease and enhanced chitinase activity. This also resulted in a stronger capacity for hydrolyzing β-1,3-glucan and improved antifungal effects (Figure 6C–E).

### 3.7. Validation of In Vitro Efficacy of Compound Bacterial Crude Protein Against Ginseng Black Spot Disease

**Figure 7:**
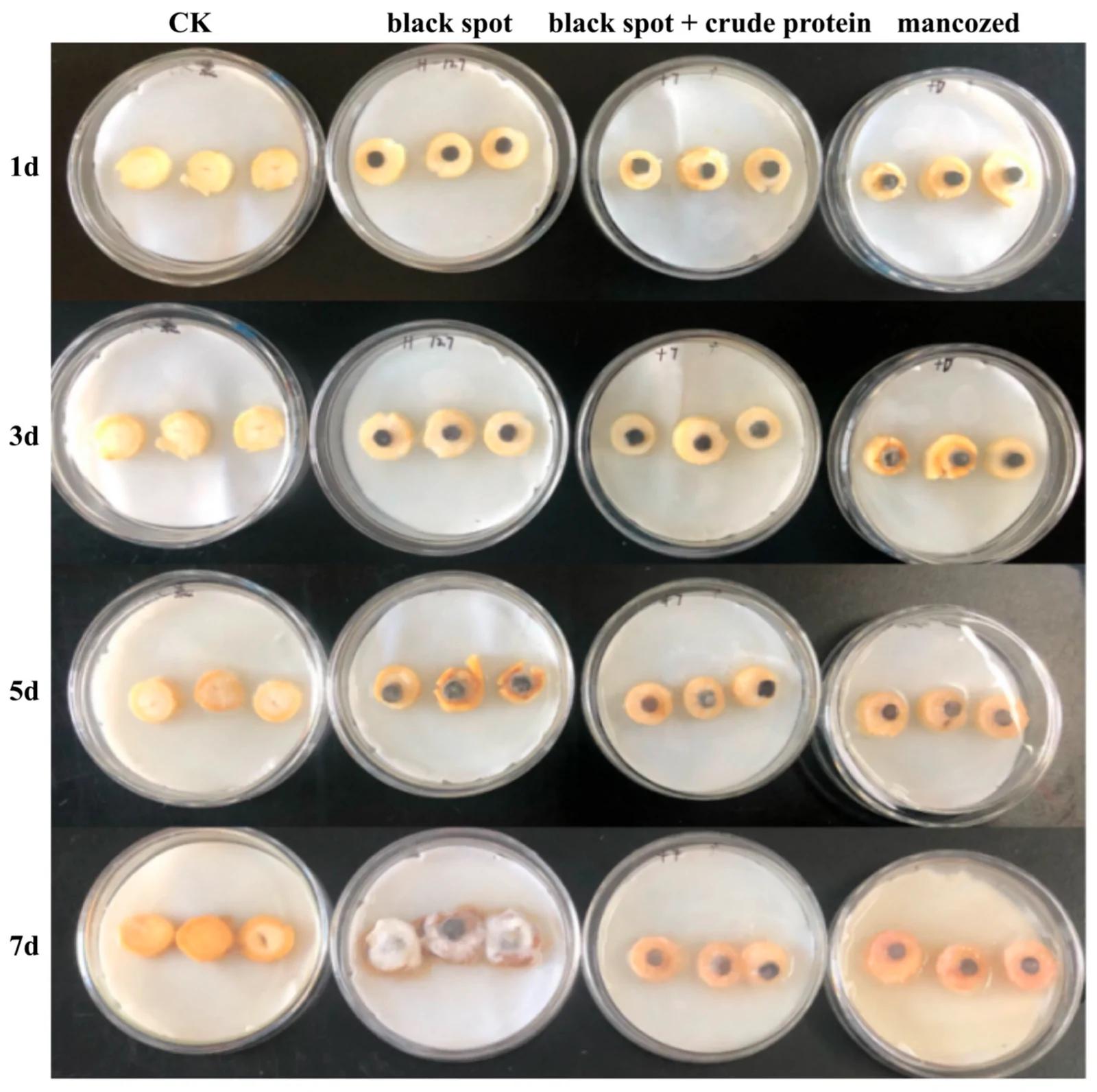
Effect of crude protein extract from the composite bacterial agent on the control of black spot disease of ginseng. CK: uninoculated blank control (sterile water); black spot: pathogen-only control (inoculated with *A. panax*, untreated); black spot + crude protein: treatment with crude protein extract from the composite bacterial agent; mancozeb: chemical fungicide positive control (70% mancozeb). Observations at 1, 3, 5, and 7 days post-inoculation were qualitative, based on visual inspection of mycelial growth and tissue decay.

Three groups of ginseng cylinders were inoculated with the ginseng black spot pathogen, while one group remained uninoculated as the blank control (CK). Following inoculation, two of the inoculated groups were treated three days post-inoculation with either the crude protein extract from the composite bacterial agent or the chemical fungicide 70% mancozeb (positive control), while the third inoculated group received no treatment (pathogen-only control) (Figure 7).

The results indicated that in the ginseng cylinders inoculated solely with black spot disease, the fungal spread was rapid over time, leading to significant rot. Brown spots emerged on the surface, and a small amount of mycelium developed by the third day. By the seventh day, the mycelium had completely enveloped the segments, resulting in rot and a softening of texture. These observations were qualitative, based on visual inspection of mycelial growth and tissue decay.

Ginseng cylinders treated with the crude protein extract from the composite bacterial agent showed no visible increase in mycelial growth by day 5, and no notable changes were observed in the subsequent stages. The visual appearance (mycelial quantity, color, and hardness) was similar to that of the mancozeb-treated group.

## 4. Discussion

This study presents a biocontrol strategy using a consortium of two *Bacillus* strains (WY17 and WY26) to enhance the antifungal efficacy and stability compared with single-strain applications for controlling ginseng black spot disease. Through systematic analysis, the two strains were found to exert synergistic inhibitory effects via complementary mechanisms involving membrane disruption and cell wall degradation. These findings provide a theoretical foundation for the development of biocontrol strategies against ginseng black spot disease.

Two endophytic bacteria were isolated from the roots of ginseng, demonstrating a strong inhibitory effect against ginseng black spot disease. Based on morphological observation, physiological and biochemical analyses, and 16S rRNA gene sequencing, these strains were identified as members of the genus *Bacillus* and designated as *Bacillus* sp. WY17 and WY26. The robust inhibitory activity of strains WY17 and WY26 against black spot disease (antifungal inhibition rate > 76%) demonstrated their strong biocontrol potential. By constructing a composite bacterial agent based on the genus *Bacillus*, we elucidated the synergistic inhibitory mechanism against the ginseng black spot pathogen. When two antagonistic strains were mixed in varying proportions, significant differences in their inhibitory effects were observed. The optimal antifungal inhibition rate occurred at a ratio of 2:1. This synergistic interaction between the two strains enhanced the inhibitory rate of the composite agent to 84.94%, markedly surpassing that achieved with single-strain treatments (WY17: 80.21%; WY26: 76.56%, *p* < 0.05). Notably, this synergistic effect peaked at a strain ratio of 2:1, contrasting sharply with the efficacy exhibited by individual strains such as *Bacillus velezensis* ML61 against black spot disease (reported up to 76%). These findings underscore the critical role of multi-strain metabolic complementarity in broadening antifungal spectra [31].

The production of antifungal active substances in biocontrol bacteria is influenced by nutritional and cultural conditions. The carbon source serves as a critical factor for bacterial biomass and the synthesis of secondary metabolites. In single-factor experiments, starch (2.5 g/L) was identified as the optimal carbon source for producing antifungal metabolites [32]. Nitrogen sources not only affect bacterial growth but also regulate the synthesis pathways of antifungal substances. Research has demonstrated that urea, as an economical nitrogen source, can significantly enhance protein content (93%) [33], while the selection of nitrate and ammonium salts influences enzyme activity [34]. Additionally, inorganic salts play a vital role in regulating metabolic pathways and physiological activities. In media containing CaCl_2_, there was a significant increase in the number of viable *Bacillus velezensis* UTB96 cells, suggesting that CaCl_2_ may promote the proliferation of biocontrol bacteria by providing essential ions such as Ca^2+^ [35]. Magnesium salts, including MgSO_4_, act as activators for various enzymes and are crucial in the production of surfactants and antifungal compounds [36]. This study employed both single-factor experiments and orthogonal trials to optimize nutritional formulations and culture conditions effectively. Through optimization experiments, an optimal medium composition (soluble starch 2%, beef extract 0.5%, CaCl_2_ 1%) was determined. The amounts of carbon sources, nitrogen sources, and inorganic salts were precisely regulated to maximize bacterial biomass, achieving an OD_600_ of 1.974 under the optimal fermentation conditions. This high biomass level indicates that the cells are in a state of active metabolism where nutrient consumption balances with product synthesis. For instance, the biomass of recombinant bacteria grown in M17 medium has been reported to be directly correlated with their metabolic activity [37].

The antifungal efficacy of biocontrol agents can be influenced by various environmental factors, which may either diminish their antifungal properties or enhance their antagonistic effects. In this study, the antifungal metabolites produced by the two strains were subjected to treatments involving high temperature (80 °C, 30 min) and Proteinase K (2 mg/mL). The results indicated that the fermentation supernatant of the composite bacterial agent retained antifungal inhibitory rates ranging from 76.76% to 78.16% under these conditions. This stability was superior to the thermal tolerance threshold observed in strains such as *Bacillus velezensis* RC218 (activity reduction >40% at 60 °C) [38].

To further investigate the substances in biocontrol bacteria that exert an inhibitory effect, we extracted the active components of these bacteria, which primarily included crude protein, crude lipopeptide, ethyl acetate, petroleum ether, and n-butanol. Antagonistic tests were performed on the extracted substances; the results indicated that the crude protein from the complex bacteria was responsible for a significant inhibitory effect among the tested extracts.

Based on the identified antifungal active substances, the crude protein extract from the bacterial consortium exhibited inhibitory effects on both mycelial growth and spore germination of black spot disease. The inhibitory effect improved with increasing concentrations, achieving an antifungal inhibition rate of 87.85% and reducing the spore germination rate to 8.63%.

Through dynamic monitoring of electrical conductivity and analysis of intracellular substance leakage, it was observed that the electrical conductivity in the crude protein treatment group significantly increased to 716.36 S/m at 5 h (3.12 times higher than the control group). These results suggest that disruption of cell membrane integrity may lead to leakage of intracellular substances such as nucleic acids and proteins, manifesting as abnormally elevated electrical conductivity. Microscopic observations revealed morphological changes in the mycelia, including lighter coloration, distortion and deformation of hyphae, reduced branching, and diminished vitality. The extent of changes observed in mycelium indicates that the crude protein extract exerts a significant inhibitory effect on pathogenic fungal mycelia. Notable shrinkage and vacuolation were evident on the surface of the mycelium. This damage to the membrane system may impede normal metabolic processes and cellular proliferation, potentially accelerating senescence and death of the fungal pathogen by hastening intracellular substance loss. The data indicated that cell membrane damage peaked after 5 h of treatment. At this time, the electrical conductivity indicators and microscopic structural observations corroborated each other, providing physiological and biochemical evidence for the antifungal action of the crude protein. This pathway of membrane damage leading to metabolic breakdown is analogous to the lipid peroxidation of membranes induced by surfactants produced by *Bacillus amyloliquefaciens* [39].

Meanwhile, the activities of protease, chitinase, and β-1,3-glucanase in the treatment group were significantly higher than those observed in the control group. Furthermore, the combined bacterial treatment exhibited greater efficacy compared to treatments with individual strains. Specifically, protease activity was found to be 2.44 times that of the control group, chitinase activity was 5.57 times greater than that of the control group, and β-1,3-glucanase activity was 2.81 times higher than that of the control group. Additionally, it was noted that an increase in the concentration of the compound bacterial agent correlated with a more rapid hydrolysis rate for protease and enhanced chitinase activity. This also resulted in a stronger capacity for hydrolyzing β-1,3-glucan and improved antifungal effects overall.

Chitinase hydrolyzes the chitin skeleton of fungal cell walls, while β-1,3-glucanase disrupts the glucan structure linked by β-1,3-glycosidic bonds [40,41,42]. The enzymatic activity of individual strains may be limited; for instance, *Bacillus cereus* Dt10 exhibits only weak chitinase activity and does not produce β-1,3-glucanase [43]. In contrast, combining different strains can significantly enhance enzyme activity. For example, specific combinations of strains (such as PBT3 and PBT13) demonstrate high activities of both β-1,3-glucanase and chitinase, respectively [44]. In this study, the crude protein-activated cell wall hydrolases from complex bacterial communities specifically degrade chitin and β-glucan in the cell walls of fungal pathogen. This “membrane-wall dual target” mode of action is more broadly antifungal than mechanisms that rely on a single antifungal approach.

To evaluate the protective efficacy of the crude protein extract, an ex vivo detached-root assay was performed using fresh ginseng segments. In this ex vivo model, the crude protein extract showed a protective effect similar to that of 70% mancozeb based on visual observations. However, since *Alternaria panax* is predominantly a foliar pathogen, the results should be interpreted within the context of this ex vivo root assay, and further in vivo studies on leaves are needed to confirm efficacy under field conditions. The effectiveness of the crude protein extract was comparable to that of 70% mancozeb in our ex vivo assay. Notably, mancozeb has been reported to reduce *Bacillus* abundance in soil by 64% [45]. This suggests that the crude protein extract may offer a more environmentally friendly alternative; however, its direct effects on soil microbiomes were not evaluated in the present study and remain to be demonstrated in future research.

This study overcomes the limitations of traditional single inhibitory mechanisms and enhances the efficiency of black spot disease control through a dual mode of action involving membrane disruption and cell wall degradation, thereby providing a theoretical foundation and technical reserve for the development of biocontrol strategies against ginseng black spot disease.

## 5. Conclusions

In this study, two endophytic *Bacillus* strains (WY17 and WY26) were isolated from ginseng roots and demonstrated synergistic antifungal activity against *A. panax* when combined at a 2:1 ratio. The crude protein extract from the co-culture fermentation served as the primary active component, exerting its antifungal effects through membrane disruption and cell wall degradation. In an ex vivo root assay, this crude protein extract showed protective effects comparable to 70% mancozeb. However, given that *A. panax* is predominantly a foliar pathogen, further in vivo studies on leaves are needed to confirm its field efficacy and environmental safety.

## Figures and Tables

**Table 1:** Physiological and biochemical characteristics of strains WY17 and WY26.

Test	WY17	WY26
Voges–Proskauer (V-P) test	−	−
Methyl Red (M-R) test	−	−
Starch hydrolysis test	+	+
Indole test	+	+
Nitrate reduction test	+	+
Citrate utilization test	−	−
Catalase test	+	+
Hydrogen sulfide (H_2_S) production test	−	+

Note: “+” indicates a positive reaction; “−” indicates a negative reaction.

**Table 2:** Orthogonal assay analysis.

Factor				
Code	Soluble Starch	Beef Extract	CaCl_2_	OD_600_
1	3	3	1	1.653 cd
2	1	2	3	1.682 cd
3	3	1	3	1.825 b
4	1	3	2	1.660 cd
5	2	3	3	1.754 c
6	3	2	2	1.943 a
7	2	2	1	1.722 c
8	2	1	2	1.974 a
9	1	1	1	1.352 e
K_1_	4.496	5.151	4.727	
K_2_	5.450	5.347	5.577	
K_3_	5.412	5.067	5.261	
k_1_	1.565	1.782	1.576	
k_2_	1.817	1.717	1.859	
k_3_	1.807	1.689	1.754	
R	0.252	0.093	0.283	

Note: Significant differences (Tukey test, *p* < 0.05) are indicated by lowercase letters in the table.

## Data Availability

The original contributions presented in this study are included in the article. Further inquiries can be directed to the corresponding authors.

## Abbreviations

The following abbreviations are used in this manuscript:

ANOVA	Analysis of variance
BLASTn	Basic Local Alignment Search Tool nucleotide
bp	Base pair
CK	Control
DBP	Dibutyl phthalate
DMAB	p-Dimethylaminobenzaldehyde
DNS	3,5-Dinitrosalicylic acid
EC_50_	Half maximal effective concentration
EC_70_	70% effective concentration
KDP	Potassium dihydrogen phosphate
M-R	Methyl Red
NA	Nutrient agar
NB	Nutrient broth
NCBI	National Center for Biotechnology Information
NJ	Neighbor-Joining
OD_600_	Optical density at 600 nm
PCR	Polymerase Chain Reaction
PDA	Potato dextrose agar
rRNA	Ribosomal ribonucleic acid
SEM	Scanning electron microscopy
SPSS	Statistical Product and Service Solutions
V-P	Voges–Proskauer
